# Menstruation and Autism: a qualitative systematic review

**DOI:** 10.1089/aut.2024.0307

**Published:** 2025-04-17

**Authors:** Rebecca Ellis, Gemma Williams, Selena Caemawr, Monique Craine, Willow Holloway, Kathryn Williams, Sebastian C.K. Shaw, Aimee Grant

**Affiliations:** 1 School of Health and Social Care, Swansea University, Swansea, Wales; 2 Aubergine Café; 3 Monique Craine Neurodivergent Matters; 4 Autistic UK CIC; 5 Fair Treatment for the Women of Wales; 6 School of Social Sciences, Cardiff University, Cardiff, Wales; 7 Department of Medical Education, Brighton and Sussex Medical School, England

**Keywords:** Autism, menstruation, experiences, periods, qualitative, neurodivergent, puberty, education

## Abstract

**Background:**

Menstruation is a biological process which can be irregular, painful, and co-occur with fatigue, social withdrawal, and increased emotions. There is some evidence to suggest Autistic people experience menstruation differently to non-Autistic people.

**Methods:**

This systematic review (PROSPERO registration: CRD42023399674) searched four databases in February 2023. We used thematic synthesis to analyse qualitative studies of experiences related to menstruation for Autistic people and people providing support to Autistic people. We also appraised the quality of studies with CASP.

**Results:**

Our thematic synthesis of twelve (eight peer-reviewed; four grey literature) sources identified differing priorities between parents, professionals, and Autistic people. Autistic people reported physical, emotional and social impacts of menstruation. Furthermore, there were reports of increased Autism-specific experiences during menstruation such as increased sensory sensitivities, burnout and anxiety, which were often unseen by those who supported them. Autistic people’s menstruation priorities were obtaining knowledge and skills ahead of their first period. However, parents and professionals focused on external behaviours including hygiene and obtaining independence in managing periods. Sources poorly described participant demographics and had a limited focus on Autistic perspectives.

**Conclusions:**

We conclude there needs to be tailored support to meet the needs of both Autistic individuals and those who support them. This should be informed by research which centers Autistic people.

## Introduction

Menstruation involves the passage of menstrual blood from the uterus through the cervix and vagina roughly every month, beginning at puberty and ending at menopause for people with wombs.^1^ In our pre-funding consultation, in recognition of the fact that many Autistic individuals are gender diverse, we received strong feedback to use gender-neutral language in our study. As such, we do so in this article. However, within the introduction the historical treatment of those assigned female at birth (AFAB) will be discussed, and sources that have used gendered language will be quoted directly.

Menstruation encompasses physical, social, and emotional experiences, the majority of which have been identified as negative by those who experience them.^2^ In a general population, there are many debilitating conditions that are triggered by the menstrual cycle, including dysmenorrhoea (painful periods)^3^ endometriosis,^4^ adenomyosis,^5^ irregular periods^6^ and premenstrual syndrome (PMS).^7^ Severe emotional changes can also occur through premenstrual dysphoric disorder (PMDD), and an exacerbation of both anxiety^8^ and depressive symptoms, typically in response to heavy menstrual bleeding.^9^ There also remains stigma and prejudice against those who menstruate,^10^ fostering shame amongst menstruators^11^ and impacting participation in social activities,^12^ plus there remains significant period poverty across the globe.^13^

There has been a long history of gender bias impacting the effectiveness of menstrual healthcare,^14^ which may be further exacerbated for those who are marginalized due to gender identity, disability, ethnicity and other demographic characteristics.^15^ In a general population, women report higher levels of pain more frequently and yet are more likely to be inadequately treated in comparison to their male counterparts;^16^ as a result of structural and internalised sexism.^17^ Recently, there has been an increased acknowledgement of the significant impacts of gynaecological conditions, which were previously neglected in research literature, including endometriosis^18^ and other conditions previously dismissed as psychogenic.^19^

Autistic people are known to experience significant barriers to healthcare, at patient, healthcare provider and system levels.^20,21^ These reduce their likelihood of being appropriately supported to access healthcare across the lifespan.^22,23^ Autism has historically been discussed within the context of boys and men,^24,25^ leading to missed or differing diagnoses of Autistic women, impacting their quality of life.^26^ In addition, historically, Autistic experiences have been framed within a medical model perspective, presenting Autism negatively, rather than the more nuanced understanding of Autism the social model encourages.^27^ Expectancy biases and gender stereotypes, alongside masking,^26^ may also contribute towards a delay in diagnosis for non-cis-male individuals^28^ and a reduced understanding of the Autistic experience in female and trans populations.^29^

Historically, there was little research on the reproductive health experiences of Autistic people, although this is a growing area of interest.^30^ For example, a recent review has found Autistic people experienced additional sensory challenges in relation to breastfeeding,^31^ and there is emerging evidence that Autistic people experience menopause differently to non-Autistic people.^25^ Moreover, published research has found that Autistic individuals experienced poorer gynaecological outcomes,^32^ impacting physical, mental and social wellbeing.^33^ Through cross-sectional research, it is suggested Autistic menstruators experience increased rates of dysmenorrhoea, PMS,^34^ endometriosis and polycystic ovary syndrome.^35^ Menses (the shedding of menstrual blood and tissue) for Autistic people can impact social activities and relationships, and increase negative experiences such as anxiety, depression and irritability.^36^ There are also additional sensory and emotional self-regulation challenges during this time and correspondingly increased meltdowns.^32^

To better understand and develop support for Autistic menstruators there is a need to collate the experiences faced by them and those who support them. To the authors’ knowledge, there has not been a systematic review specific to experiences of Autistic menstruation conducted previously.

## Materials and Methods

### Aim

Our aim was to undertake a systematic review with meta-synthesis to better understand the views, experiences and impact of menstruation on Autistic people.

Our research is reported in line with PRISMA reporting standards.^37^ A copy of the completed PRISMA checklist is available in the appendix (supplementary material 1).

### Community Involvement

Our review was led by an Autistic researcher (RE) and benefitted from the involvement of two other Autistic researchers (GW, AG), an Autistic primary care doctor (SS), and four lay Autistic people with relevant lived experience (SC, MC, WH, KW), all of whom are authors. Our thematic synthesis involved the community members at each phase of the data analysis, where ideas were discussed, reshaped, and solidified. The discussion for this article was built collaboratively by the full research team.

### Search Strategy

We identified our search terms by hand searching keywords of relevant papers and terms used in systematic reviews focused on menstruation in a general population.^38–40^ Autism related search terms were generated using similar reviews.^31,41^ The search strategy involved two strings of terms relating to Autism and menstruation:

(i) Autism; Autism, Autism Spectrum Disorder, ASD, Autism Spectrum Condition, ASC, Neurodiverse, Neurodivergent, Autistic Disorder, Child Development Disorder, Asperger’s, Neurodevelopmental Disorder, Neurodevelopmental Condition, and
(ii) Menstruation; Menstruation, menses, menstrual cycle, menstruators, ovulation, ovulating, menarche, menstrual period, follicular phase adrenarche, premenarche, catamenia, amenorrhea, premenstrual dysphoric disorder, PDD, premenstrual syndrome, premenstrual exacerbation, PME, PMS, menstrual age, menarche age, age at menarche, menarchal age, menarcheal age, puberal haemorrhage, puberal hemorrhage, menstrual haemorrhage, menstrual hemorrhage, period products, menstrual products, dysmenorrhea, menstrual pain, heavy menstrual bleeding, painful menstruation, period pain, menorrhagia, oligomenorrhea, adenomyosis.

Following the publication of our protocol (PROSPERO registration number: CRD42023399674), we searched four electronic databases to reflect a range of academic disciplines; Medline (via Ebscohost), APA PsychInfo (via Ebscohost), CINAHL (via Ebscohost) and Web of Science (via Clarivate). Database searches were limited to the English language, full text and by publication date (2013 to present). Searches were undertaken in February 2023. Grey literature was searched for using citation tracking, additional searching through Google Scholar and the Swansea University online library, plus other search engines. One systematic review, which focused on educational interventions about menstruation,^42^ was searched for additional sources, but none were identified. A sibling paper focusing on menopause is registered on PROSPERO (registration number: CRD42023450736) and is in progress, so was not a topic covered in this review.

Sources were screened against the following criteria:

#### Inclusion criteria

- Population: Autistic people (including self-identified) and those who support them, including health professionals, family, and friends.
- Context: no limitations.
- Phenomenon: Views and experiences of menstruation.

#### Exclusion criteria

- Articles which do not focus on the population described above and menstruation.
- No qualitative data (note: open text survey responses, commentaries and grey literature containing personal experiences were included).
- Published prior to 2013.
- Not English language.
- Full text not available.

### Screening and Data extraction

Two reviewers (RE and AG) independently reviewed all titles and abstracts identified against the inclusion and exclusion criteria. The full texts of potentially relevant studies were reviewed independently by two reviewers (RE and AG). Inconsistencies at both stages were resolved through discussion, and it was not necessary to involve a third reviewer. Following this, contextual information, including findings to be included in the thematic synthesis, were extracted from sources using a structured summary tool (see Supplementary material 2).

### Quality Assessment

We evaluated all studies for quality using the CASP qualitative checklist^43^ by one researcher (RE). A sample of 10% was independently appraised by a second reviewer (GW). Any disagreements were discussed and resolved at this time. Each study was provided with an overall assessment of quality: high (7-10), medium (4-6) and low (0-3), as has been used in other qualitative systematic reviews.^44^

### Data Analysis

The thematic synthesis approach was adopted following Thomas and Harden’s^45^ framework. This process included an inductive multiple-stage approach, with stage 1 and 2 compromising the coding of the text and development of descriptive themes and stage 3 being the generation of analytical themes.^45^ Thematic synthesis is aligned with critical realism,^46^ which was adopted in this review. A critical realist perspective considers unobservable constructs, such as social contexts and perceptions, which in turn impact findings in the ‘observable’ world.^47^

As part of the thematic synthesis, three online meetings (total 7 hours), broadly reflecting the three Thomas and Harden (2008) stages,^45^ were held between May and July 2023, to: discuss the themes initially developed by RE, generate analytical themes and develop discussion points. These meetings were made accessible to meet the needs of the lay members involved. We describe our process of building an accessible space with this group elsewhere.^48^

### Stages 1 and 2: Coding and Development of Descriptive Themes

Structured summaries of each source, containing information about the study or source as well as all results related to Autism and menstruation, were uploaded into NVivo 13.^49^ Findings were inductively coded line-by-line by one researcher (RE). A second researcher (GW), independently inductively coded the first five sources, identified by a random number generator, to ensure codes with similar meanings were developed. Multiple lines were attributed to more than one code. Following discussion, minor changes were made to the descriptive coding framework, the remaining 7 items were coded by RE, and a written document and thematic map were produced and shared with the full research team for discussion.

### Stage 3: Generation of Analytical Themes

We then generated analytical themes, with coding iteratively expanded and collapsed upon. Written work and thematic maps were updated following each meeting.

## Results

### Studies Identified

**Fig. 1:**
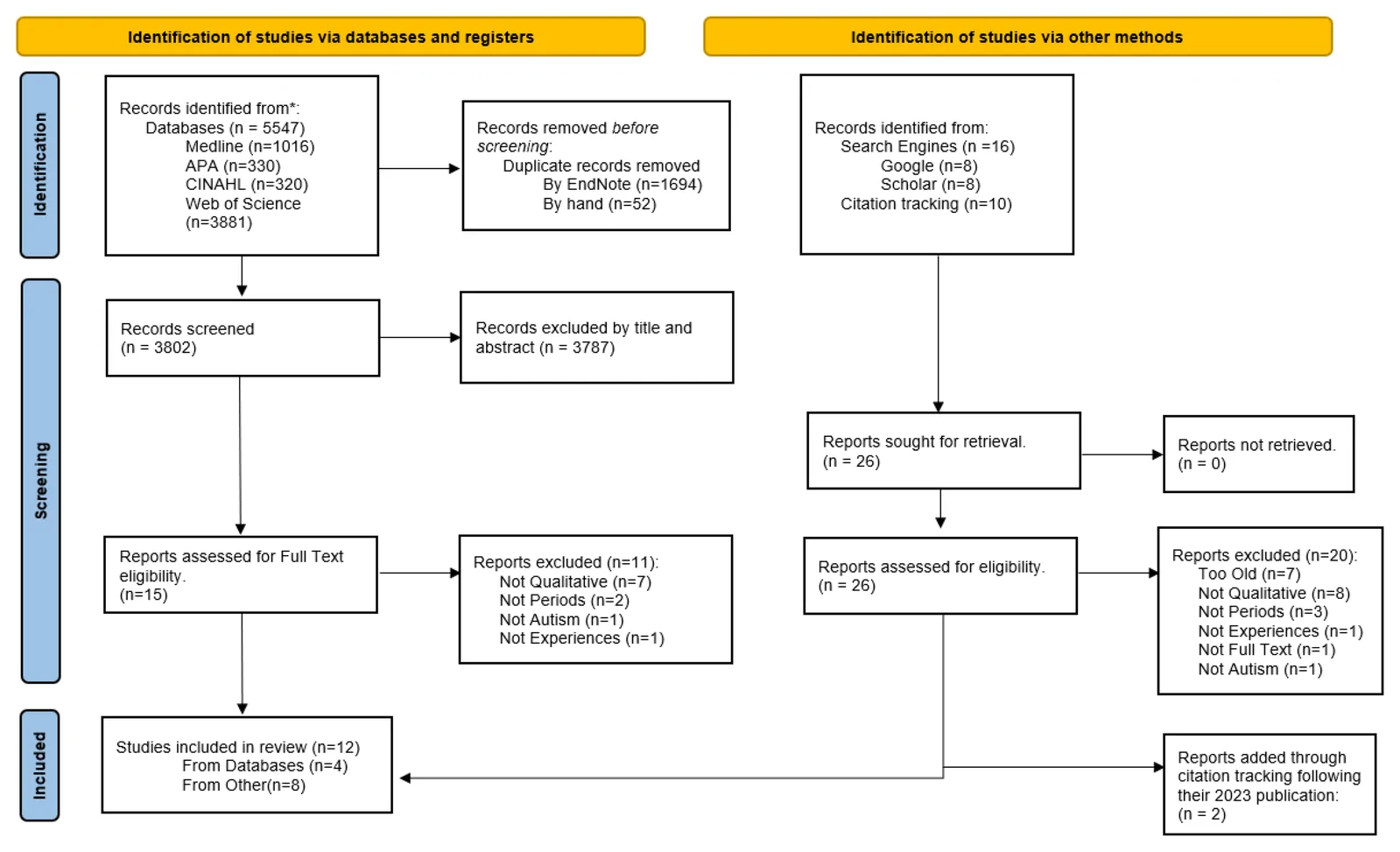
Flow diagram showing the selection of reports included in the review.

We identified 3,802 unique records through database searches (see Figure 1). Fifteen papers were assessed for full text eligibility and four papers were included through the database search. Two further peer reviewed papers were found through search engines. Two additional peer reviewed studies, which were published following our database search, were identified from citation tracking.^50,51^ Four pieces of grey literature were identified: an article on an advocacy website, a blog entry, a business report, and a master’s dissertation. In total, we included 12 sources in the review.

### Characteristics of included Studies

Our sources included participants from the United Kingdom (UK) (n=4), the United States of America (USA) (n=3) or Australia (n=3), with others based online (n=3) (see Table 1). Most sources did not have a specific setting (n=9). Participants were recruited through social media (n=4), subscription-based organisational correspondence (n=3) and schools (n=2). Described participants included: Autistic people (n=195) of which the majority were adults (n=172), parents of Autistic children and young people (n=76), educators of Autistic people (n=10) and other non-Autistic people (total n=134). Three papers gained input from more than one participant group,^32,50,52^ the remaining papers included contributions from either Autistic people (n=5) or parents and professionals (n=4). The most frequently used data collection method was semi-structured interviews (n=7), with thematic analysis the most used data analysis strategy (n=7). There were mixed uses of descriptive language across the sources, with seven using identity-first language and five using person-first language. When assessed against the CASP qualitative critical appraisal tool,^43^ all nine empirical sources were identified as high quality (see Supplementary material 3). Three sources were not included in the quality review because they were not empirical in nature^53,54^ or did not describe their research process.^55^

### Thematic Synthesis

**Fig. 2:**
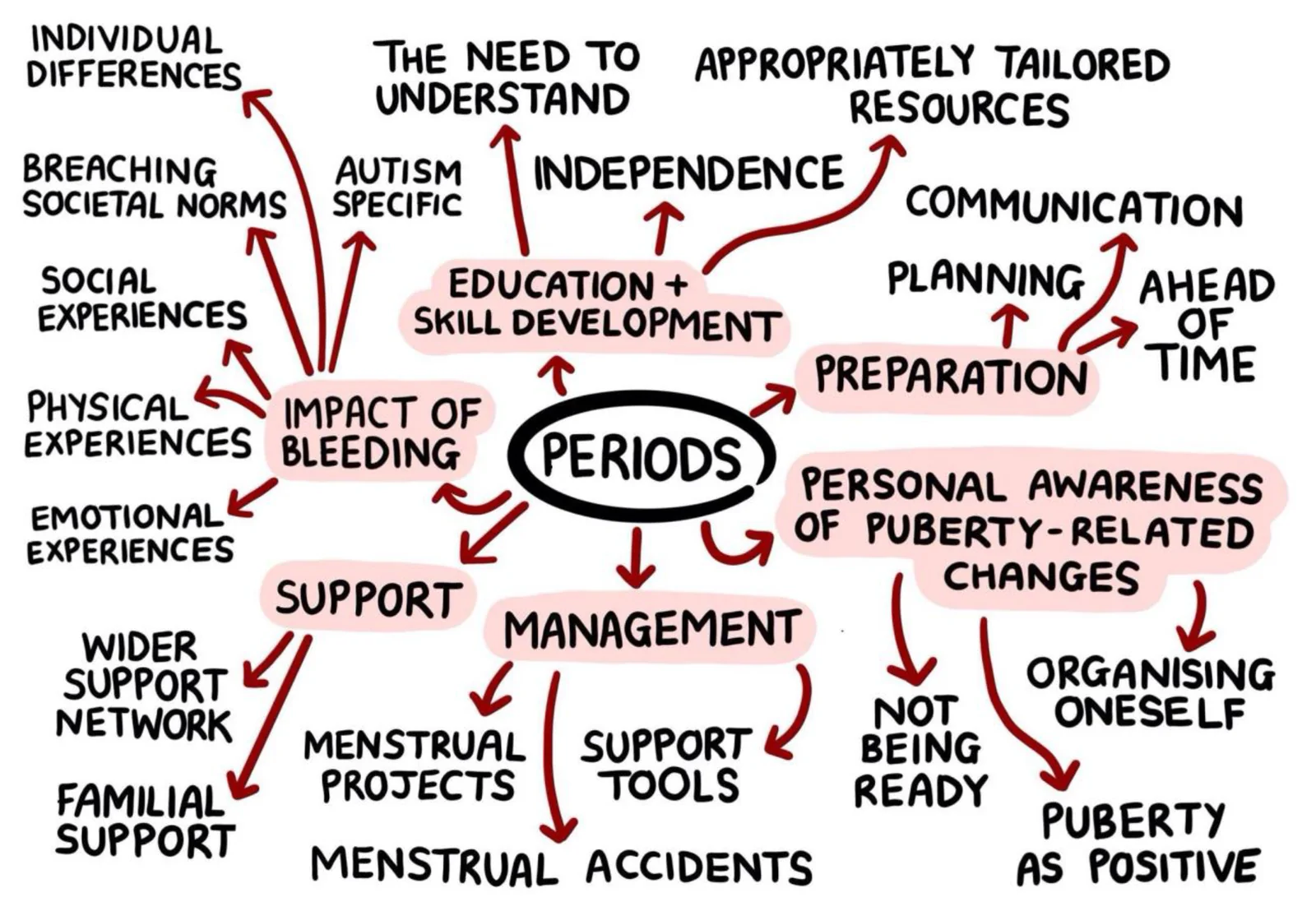
Graphical representation of themes.

We identified six themes from the included literature: *education and skill development, preparation, personal awareness, management, impact* and *support*, which are outlined below. Sub themes are underlined within the narrative for emphasis. A graphical representation of the themes can be seen in Figure 2.

Following our thematic synthesis, we note which voices were represented and differences in our analysis between groups. Table 2 shows which themes are covered in which sources by participant group.

### Theme 1: Education and Skill Development

This theme was split into three sub-themes. **The need to understand** biological knowledge was emphasised by all groups of participants.^32,50,52,53,55,60^

““I think a lot of issues could be addressed through education too, for example I hardly know the basics of my own cycle and what I know is from basic biology at school… Especially in school, there was nothing and I needed that. It was taboo at school, and it was too awkward to talk about it so I learned nothing, and I could have benefited from learning.” (Non-Autistic participant 1).” ^50^

Similarly, addressing the taboos surrounding periods plus emphasising that menstruation was normal and something one could speak about, was also discussed by both Autistic^32,50,52,60^ and non-Autistic contributors.^32,50,52,55^

““I think culturally, I think especially people without uteruses who haven’t experienced it, they tend to not really believe that pain. Especially, if that person looks like a woman. Women are often disbelieved by people or seen as over-exaggerating, so I think it’s probably not taken as seriously.” (Autistic participant 17).” ^50^

For education providers and parents, there was a focus on providing **appropriately tailored resources**.^32,52,55,57,60^ It was clarified that knowledge transfer should be through resources which used appropriate language^32,50,55,57^ and were suited for current understanding.^52,57^

“…When I saw opportunity for [a] video, I thought [since] she’s always been such a visual learner, I thought this is really great, because the more we can discuss it then she can get more comfortable with it. Everyone presents it in a little different way, and I thought she picked up information from each source a little bit differently.” (Parent 8 (46)) ^52^

There were comments made, the majority from parents, suggesting Autistic menstruators should prioritise “*basic, functional skill*” development,^57^ and work towards managing oneself, with the goal of reaching **independence**.^52,56,57^

““I’m confident by the time she’s 19 [in 8 years], she’ll be pretty good. I mean I don’t know how fully independent she’ll be, but I’m confident she’ll be able to, with her watch [a vibrating watch, set to vibrate at set intervals, to remind the daughter to go to the toilet to change her pad], take herself to the loo and change her pad” (Parent).” ^57^

Reflections on independence, all from non-Autistic contributors, included general reflections on the level of support required by some children in relation to periods^52,57^ and setting independence and menstrual management as an end goal to skill development.^56,57^

### Theme 2: Preparation

This theme related to the time before and during the onset of menarche (the first menstrual period) and has three sub-themes. Considering **planning**,^50,52,55,60^ Autistic people discussed feelings of shock and anxiety when their period started, often regardless of how well-informed they felt,^50,52,55,60^ with some alluding to interoceptive differences as a contributing factor.

“So, even though I’d had those sessions where they give you the biology side of it like, this is what happens, it didn’t really connect into practical for me. I understood the biology, it was just that I didn’t realise that was what it was, which seems stupid now.” (Autistic participant)^60^

There was also a mention of distress for Autistic people who wished their periods were more predictable.^52,60^ It was emphasised that Autistic children and young people benefitted from frequent and open **communication** about menstruation.^52,56,57,59,60^

““Then after she had that discussion, we talked about it here and there. I actually let her ask me questions about it, rather than me initiating it, and I think that also helps. I could see how it could be very scary though for both the mother and child, but for me I didn’t get that feeling just because I had a lot of support and I had a pediatrician that took the time to spend time and talk about that.” (Parent 2)” ^52^

Also, both Autistic and non-Autistic contributors recommended that knowledge and support items should be provided **ahead of time**.^32,52,53,55,60^

“Several autistic respondents noted specifically that “it was helpful to know beforehand that I wasn’t dying” (Autistic participant 7) from the bleeding and/or pain.” ^32^

Although, there was some disagreement as to who should provide this (the parents, educators^52,57^ or medical professionals^52,57^) and when (upon onset of menstruation^57^ or beforehand^57^).

### Theme 3: Personal Awareness of Puberty Related Changes

The “Personal Awareness of Puberty Related Changes” theme was split into three sub-themes.

There were four sources in which **puberty was discussed positively**.^51,52,57,60^

““Well, once you get older you start to feel more mature, and your body’s changing and you start to notice something different about yourself.” (Daughter 5)” ^52^

However, the majority of contributions discussed negative emotional reactions to biological changes. Often Autistic people reported feelings of **not being ready**^50,52,55,59,60^ by questioning what was happening^50,52,55,57,59,60^ and acknowledging remaining gaps in their knowledge.^32,50,52,55,56,60^

““I feel like there is a link between the Autism and the way that I experience my period, because it was really distressing for me when I started developing… it was so distressing for me that change. I had no context to it.” (Autistic participant 11)” ^50^

Some Autistic participants were unsure as to whether they would experience puberty in a similar way to their neurotypical peers, if at all. ^52,55,60^

“Though I logically knew this as a fact of life, I felt so removed from others that it was shocking and traumatising when I got my period for the first time. I remember my mother, asking me what I’d expected, and I remember telling her that I thought I might be “like an alien” − a genetic anomaly that was exempt from such things.” (Autistic contributor) ^55^

There were several reflections about **organising oneself** ^50,52,53,55,58,60^ with a disconnection felt by several Autistic contributors between themselves and their body,^50,52,53,55,60^ impacting their ability to judge when their periods were due,^52,53,55,58^ and additional precautions they took to rectify this.^52,60^

“My period is very irregular which I find distressing… I never know when it’s going to come so I can’t properly prepare myself for it.” (Autistic contributor) ^55^

““She has taken ownership of it and is very vigilant, almost sometimes a little too vigilant with checking her pads…” Parent 8” ^52^

### Theme 4: Management

The theme of “Management” includes three sub-themes. There were eight sources in which specific **menstrual products** were discussed,^32,51–53,55–57,60^ including tampons,^52,53,55,57,60^ pads,^32,52,53,55,57,60^ menstrual cups^53,60^ and period pants.^60^

“Tampons are, in my opinion, better than pads, but they’re still not without their flaws. Anyone who told me that you could pee while having a tampon in must have a magic trick or their anatomy might be slightly different which enables this to happen, but I found that the stream would somehow come into contact with the string, which would then absorb into the tampon itself and render it completely useless.” (Autistic contributor) ^53^

The usage and disposal of these products were also described with all parental contributions reflecting on incorrect usage,^52,56^ for example children having an inflexibility with trying new menstrual products^52^ and their frustration with this:

““She’s more worried about leaking, and so she will at times, we’ve discussed, you know I’ve found her wearing up to three pads at a time, and so we talked about how instead of doing that I could get like overnight pads, you know there was a different product, and so that helped. It was more of an anxiety about having an accident or leakage, than comfort. I was kind of surprised how she could walk around with three pads! Would’ve drove me nuts!” Parent 8” ^52^

““I’m frustrated that she’s so rigid. She won’t. I tried to show her… You know like even [with] tampons, it would make your life so much easier. But, she just doesn’t care. So, I’ve tried different things. Especially with the tampon, that didn’t get me anywhere. I think the sensory [thing is an issue]. I think the thought of her sticking something in her is just beyond what she can deal with.” Parent 9” ^52^

Eight sources spoke of **support tools**^32,50,52,53,55,57,59,60^ that Autistic people, their families and carers employed, most frequently the use of hormonal management, specifically the contraceptive pill.^50,52,55,57,59,60^

““I took her to the [doctors], we had to talk about it when I took her to the doctor to get her on the pill. Kind of explain why, why you’re taking this pill and you know, to make [her period] less and there are other options too to make it even more less. I wanted to start with this one. But you know it’s, it’s more just you know, just maintenance. It’s maintenance.” Parent 9” ^52^

There was also a discussion surrounding **menstrual accidents**,^32,52,53,55–60^ both reflecting on past incidences^52,53,55,60^ and either making changes to ensure they happen less frequently or reporting how accidents lessened over time.^32,52^

“A lot of it for [her] is awareness, and so now that she’s aware that ‘Whoops! You can have an accident’, or you could like have it through your clothes, um she’s more conscientious of what she wears, what color she wears or sitting on towels during like a heavy day. She does that all by herself now, I don’t have to do that… Parent 2”^52^

### Theme 5: Impact of bleeding

The theme of “Impact of bleeding” was split into six sub-themes. There were comments remarking on the **individual differences**^50-52,54,55,57^ of Autistic menstruation, with an acknowledgement that there were different “normals” for different people,^32,50,52,57,60^ including with how pain is perceived and communicated.^50,52,57^

“I feel like when I experience things in my body, I experience it quite intensely… things like period pain and all of that, I think can have quite an impact on my mood and my anxiety because some-thing is happening within my body and its sort of out of sorts, I struggle to remove my focus from it… it becomes a focus for me until it is done. (Autistic Participant 16).”^50^

Autistic menstruators were directly compared, both positively^56^ and negatively,^52,56,58^ to their neurotypical peers and siblings, mostly by parents.

“So yeah I would like to stop doing that…And I do see my friends and their (neurotypically developing) girls seem to take all that responsibility onto themselves and it would be nice not to have to do it but it’s the way it is (Sharon, mother).” ^56^

Eight sources spoke of Autistic children **breaching societal norms**,^32,50,52,56–60^ including parental expectations^58,59^ and those which were gender specific.^50,52,59^ Five sources discussed **social experiences**,^50,52,54,57,60^ with concerns about “missing out” from all participant groups,^50,52,57,60^ with two contrasting comments from Autistic people about actively choosing to reduce activities during their period.^54,60^

““She’s expressing concerns, like she can’t go to track practice or basketball because ‘I have my period’ and we talked about that you can’t stop living when you have your period and so you can [still] do those things, and just you know make sure that you’re fully prepared. If I let her, she wouldn’t have gone, but we made her go and then she realized that she didn’t have to let it stop her. But she’s more resistant sometimes when it comes to physical activity or going places, she’ll be like ‘Oh well, I can’t go out with you guys because I have my period’ and I’m just like ‘Oh well, it’s portable, so let’s go!’ So just walking her through that.” Parent 8 (46)” ^52^

“I try to avoid any social events during my PMS because social interaction is just too exhausting then. If it was possible, I would avoid any events at all, except for lying on the sofa while watching TV and eating chocolate.” (Autistic contributor) ^54^

The most frequently reported **emotional experiences**^32,50–52,54,55,57–60^ included: mood swings,^32,50,52,55,59,60^ anxiety^32,50,52,54,55,60^ and increased emotional experiences such as sadness^32,50,52,55,57,60^ and embarrassment.^50,52,55,58,60^ **Physical experiences**^32,50–52,54,55,57,60^ included pain and discomfort, PMS and the impact of menstruation on co-occurring conditions, such as epilepsy. **Autism specific**^32,52,54–60^ reflections differed according to stakeholder group.

Autistic participants reflected on menstrual-related experiences which are inherently related to being Autistic, including increased meltdowns,^32,52,54,55,60^ overwhelm,^50,52,55,60^ or burnout,^55^ reduced capacity to mask,^32,52,54^ increased sensory sensitivities^32,50,52,54,55,60^ and differences in executive functioning.^32,50,52,54,55^

““I think having periods as an autistic person is really overstimulating… it takes so much energy… period pain, and the bloating, and the back pain, and the breast pain, and the joint aches, it’s just super overstimulating. (Autistic Participant 5)” ^50^

Parental reports of these experiences were significantly fewer. Instead, parents often focused on perceived attitudes and behaviours, attributing these to their child being Autistic,^56^ including their child’s logical and factual attitude,^52^ and differing levels of what parents considered to be appropriate personal hygiene.^52,56,58,59^ Parents spoke of their child’s inherent vulnerability,^52,56–58^ expressing concern about their indiscretion and reliance on others.^56,57^

““Because misinformation is bad for a Neurotypical kid, it’s really bad for our kids. You know, because that makes them a lot more vulnerable.” Parent 3” ^52^

There were a mix of comments with regards to the future, all made by parents, with parents feeling excited,^52^ worried^52^ or mixed about their child’s future.^52^ Multiple non-Autistic reports were of a judgmental or infantilising nature,^52,56–59^ often remarking on how their child’s actions impacted themselves^52,56,59^ or others^52,59^ negatively.

“Some additional worries were in regard to appearance, embarrassment and lack of advocacy skills. “[she has] no interest in appropriate dressing”; “she wears ill-fitting clothes”.” (Parents).^58^

### Theme 6: Support

The theme of “Support” consists of two sub-themes. Most comments were regarding **wider support networks**,^32,50,52,53,55,57,60^ including school and educators,^32,50,52,53,55,57^ medical professionals,^50,52,55,57^ extended family,^32,52,60^ other menstruators^32,50,52,57,60^ and other Autistic people,^32,60^ which were made by parents and professionals. Two references were made by an Autistic person gaining support from their peers,^32,60^ with others being unsure as to whether they could discuss such a topic in their social circle.^60^

““So, it was kind of like ‘okay yeah I can now talk about it’ then realised that you can’t talk about it… I wanted to ask my friends, the little friends I had like what do I do about it, and they were like ‘you don’t talk about it’…” (Autistic participant)^60^

Autistic people’s comments were primarily about knowing who to talk to and what to say,^32,52,60^ with an emphasis on knowledge being provided in advance, such as help with preparing a “*script for what to say to a nurse…*” (p.4290).^32^ Contrary to this, educators debated the most appropriate time to introduce these topics to students.^57^ In terms of **familial support**,^32,50,52,55–57,59,60^ Autistic people reported positive incidences of both parental practical support^52,56,57,60^ and taking the lead.^52,55,57,60^ Parents reported feeling proud and confident about their child,^52,57^ having to take a more active role in their child’s care practices through prompting,^52,56,59^ and relying on external support networks.^52,57^

## Discussion

Our review highlights that there are Autism-specific experiences of periods which may vary compared to non-Autistic experiences. However, there is a discrepancy in how these are discussed between Autistic people and non-Autistic supporters. Autistic people often employed a value-neutral perspective when discussing their Autism-specific experiences.^61^ In contrast, parents often employed a medical model perspective, attributing their child’s logical thinking and practical approach to menstruation, somewhat negatively, to their child being Autistic, often comparing this to neurotypical peers and siblings. In doing so, they reinforced the neurotypical bodymind as the “norm”,^62^ and sometimes displayed frustration at these differences.^63^ Three studies detailed parental foci on the progression of their child towards independently managing their menstruation, whereas no Autistic participant spoke of this − a phenomenon identified elsewhere in the literature.^64^ Viewing Autistic menstruation experiences through non-Autistic models of reference may negatively impact research and support provided.

We identified a perceived vulnerability in Autistic people among non-Autistic adults (see: Theme 5, “Autism Specific” sub-theme) as demonstrated elsewhere in the literature.^65^

Furthermore, there was very little focus as to how professionals and parents could foster empowerment, teach consent, and encourage decision-making in Autistic people. Research highlights an assumption that menstruation is something Autistic people cannot cope with^66^ and that sexual education is less important for Autistic and disabled students.^67^ Parental attitudes towards their child’s menarche also socially reinforce the stigma surrounding periods,^68^ especially for disabled people who experience multiple layers of discrimination.^66^ Unfortunately, the perceived vulnerability of Autistic individuals could become a self-fulfilling prophecy, as the infantilising of Autistic and disabled young people, has led to minimal tailored support, as highlighted by parents in this review and in other research literature focused on Autistic people.^69^ Within this review, most of the comments surrounding support were made by parents and professionals (74%). Recently attempts have been made to improve healthcare practices for Autistic people,^70^ although to date these are not yet effective in removing the known inequalities.^71^

For Autistic people, “normalising” period pain invites danger, due to the increased prevalence of dysmenorrhea and premenstrual syndrome^34,72^ as well as alexithymia,^73^ and potential differences in pain sensitivity.^50^ Alongside this, communication differences in quantifying pain,^74^ and understanding internal pain indicators,^75^ may likely reinforce reduced support-seeking behaviours,^50^ in a context where healthcare is often inaccessible to Autistic people.^76^ There is often a societal assumption that knowledge is obtained through social osmosis^77^ with a perceived shared understanding between parents and children,^78^ which this synthesis challenges in an Autistic population, where parents under-described physical symptoms compared to Autistic menstruators. Comments from professionals, parents and other non-Autistic contributors were often limited by what they were able to see, based on the external behaviours of their children, with less focus and understanding of emotional, internal or mental states.

There is a notable difference between Autistic people having a strong biological understanding of periods (see: Theme 1, “the need to understand” sub-theme) and applying this information to their own experiences (see: Theme 2, “planning” sub-theme), as has been identified in relation to birth^79^ and breastfeeding where interoceptive differences were identified as a barrier.^31,80^ Education about menstruation typically focuses on biological knowledge and related products,^81^ rather than teaching the application of knowledge given to one’s own circumstances and experiences. Not meeting hygiene, social, or gender expectations, was often viewed by non-Autistic adults as reflecting a lack of interest on the part of the Autistic person,^56^ rather than an absence of holistic support.

There were few reports of Autistic people discussing menstruation with their peers (n=2). However, general population studies show that social learning is important.^82^ Menstruation is shaped by socio-cultural contexts,^2^ including values and social conventions.^83^ Therefore, Autistic children are perhaps at a disadvantage compared to their neurotypical peers who rely on the ‘hidden curriculum’ of unwritten cultural, social, and academic messages communicated, explicitly or implicitly, to, or between, learners^84^ for knowledge transfer. Furthermore, social exclusion for Autistic young people creates fewer opportunities to establish a framework of reference to understand ‘normal’ period experiences, impacting their help-seeking behaviours.^50^ This may also result in self-silencing behaviours,^85^ in which people feel unable to have these conversations due to lower sexual awareness, social anxiety,^86^ menstruation stigma^71^ or the fear of being “*dismissed, humiliated or invalidated*” (p.7).^55^

### Recommendations

Multiple participants discussed the need for tailored support. Building on these recommendations, our thematic synthesis identified additional recommendations. Any support developed should be created for Autistic young people, Autistic adults and their supporters, with each group’s needs reflected in the content and format, including accessibility to those with learning disabilities. Resources should be holistic in nature, including information on menstrual supplies, facilities, supportive environments, and accessible healthcare^87^ and modified to fit Autistic communication preferences,^88^ such as using age-appropriate pragmatic language^89^ and visual elements.^90^ Being provided with materials at home, school or otherwise, ahead of time, aligns with initiatives to end period poverty and achieve period dignity.^13^ Supporters should be helped to be prepared for the potential questions Autistic children will have on Autism-specific menstrual experiences, so they are more able to provide the knowledge that the young person needs. Any resources developed should be neurodiversity-affirming, and co-produced using established frameworks,^91^ reducing both the pathologising of Autism and the stigma surrounding menstruation. Autistic peer support and the ‘hidden curriculum’ are both topics worthy of further exploration within the menstruation research literature. Further research should also include describing the way in which consent for health care procedures, such as beginning a contraceptive, is obtained in Autistic young people. All future research should centre Autistic people to address current gaps in understanding.

### Strengths and limitations

This review is the first of its kind to collate the views and experiences of Autistic people, and those who support them, with regards to menstruation. The research team consisted of Autistic individuals, including lay co-researchers, who were involved at each stage of the synthesis.^45^ We limited our database searches to the English language and papers published in the last decade, which may have impacted our coverage of the available research literature. In addition, demographic information was described poorly in many of the papers, including regarding co-occurring conditions and learning disabilities. In addition, most of the Autistic participants, regardless of age, reflected on their first period and teenage years, largely failing to demonstrate older Autistic adults’ experiences of menstruation. Finally, there was limited information on who was involved in the development of educational interventions, including the extent of any Autistic involvement within Healthcare and Educational institutions.

## Conclusion

Our systematic review of Autistic experiences of periods, has highlighted discrepancies between the needs and priorities of the Autistic and non-Autistic participants. Currently there is a parent-dominated narrative with fewer in-depth Autistic contributions, minimising how much is understood of the Autistic menstrual experience, especially those which are internal, individual experiences, including pain. Despite steps being taken in recent decades to address the gaps in knowledge in gynaecological care, further research is needed to understand the gynaecological experiences of Autistic menstruators, including those from underserved communities, such as those with learning disabilities. This knowledge would aid in the development of person-centered interventions for both Autistic people, their families and carers.

## Supplementary Material

Supplementary Material

## Figures and Tables

**Table 1:** Summary of included studies

Author (Year) Country	Setting	Aim	Recruitment	Participants	Factors Impacting Results	Data Collection	Data Analysis	ND Approach	Funder	Quality Rating
Cridland et al. (2014)^56^ Australia	Not Specified	To investigate experiences of adolescent Autistic girls.	Through local schools and community groups.	Mothers (n=5) Daughters (n=3)	Formal diagnosis	Semi- structured interviews	IPA	Person-first language Use of “ASD”	Not Specified	Y (10) N (0) CS (0) H
Cummins, Pellicano and Crane (2018)^57^ UK	Not Specified	To explore the puberty experience of Autistic girls.	SEN school pupils.	Parents (n=10) Educators (n=10)	Minimally verbal Autistic girls with IDs.	Semi- structured interviews	Thematic analysis (Braun and Clarke 2006)	Identity-first language	Not Specified	Y (8) N (0) CS (2) H
Eriksen, W.T. (2016)^52^ USA	Not Specified	To explore the experience of menses for Autistic females and families	Autism research databases	Mothers (n=10) Daughters (n=10)	Daughters “…able to read English at a 4th grade level”	Semi- structured interviews	Thematic Analysis Braun and Clarke (2006)	Person-First Language ‘On the spectrum’ also used	University of Pennsylvania School of Nursing Office of Nursing Research	Y (9) N (0) CS (1) H
Gray and Durand (2023)^50^ UK	Not Specified	To explore experiences of period pain and treatment uptake in allistic and Autistic menstruators.	Social media (18+)	Allistic (n=20) Autistic (n=17)	Experience of period pain required Self- identifying accepted	Semi- structured interviews	Thematic Analysis Braun and Clarke (2019)	“…menstruators on the autism spectrum” (p.10) Identity-first language.	None associated	Y (10) N (0) CS (0) H
Grove et al. (2023)^51^ Australia	Not Specified	To understand the everyday experiences of autistic women and gender diverse people and how these impact on their health and wellbeing	Social media, and related organisations. (18+)	Autistic women and gender diverse people (n=31)	Aged 21-63. White European (77%) Medical diagnosis (83%)	Semi- structured interviews	Thematic Analysis Braun and Clarke (2019)	“Autistic led study”	UTS Chancellor’s Research Fellowship	Y (10) N (0) CS (0) H
Jones and Datta (2022)^55^ [Online]	Not Specified	To tackle menstrual taboo and stigma	Not Specified	Not Specified	Swedish business Autistic authors	Not Specified	Not Specified	Identity-first language	INTIMINA	N/A
Kendall (2023)^54^ [Online]	N/A	N/A	N/A	N/A	Autistic and ADD authors	Article	N/A	Identity first language	N/A	N/A
Mademtzi et al. (2018)^58^ USA	Child Study Center Developmental Disabilities Clinic.	To determine educational and therapeutic needs and activities for Autistic girls and their families.	Contacted those previously diagnosed at clinic.	Parents of Autistic children (n=40)	No exclusion criteria for: age, level of support or co-occurring conditions.	Focus Groups (n=5)	Thematic Analysis	Person-first language	*Hilibrand Fellowship and Marilyn and Jim Simons*	Y (9) N (0) CS (1) H
Navot, Jorgenson and Webb (2017)^59^ USA	Seattle Children’s Research Institute.	To investigate the maternal experience of raising an Autistic daughter.	Identified from prior interest in research participation	Mother daughter dyads (n=11)	Medically diagnosed “functional and fluent verbal language”.	Semi- structured interviews	*“…An interactive process commonly used in a naturalistic inquiry.”* (p.538)	Person-first language “*symptom presentation*” (p.537)	*“Supported by the Crown Family Foundation”* (p.543)	Y (9) N (0) CS (1) H
Ross (2017)^53^ [Online]	N/A	N/A	N/A	N/A	Opinion piece	Blog	N/A	Identity first language Autistic women; autistic girls.	N/A	N/A
Steward et al. (2018)^32^ UK and Australia	Not Specified	To understand both what Autistic people wanted to know about menarche, and to discuss autism-specific experiences	Website and social media	Autistic (n=123) Non- Autistic (n=114)	Autistics, parents, professionals and siblings	Online Survey	“Thematic analysis (Braun and Clarke 2006).” (p.4288) Tools used unspecified	Identity-first language Autistic researchers involved	*Pears Foundation (RS) grant and a Philip Leverhulme Prize 2015*	Y (9) N (0) CS (1) H
Whiting (2020)^60^ UK	University of Bolton	Explore the experiences of Autistic females including puberty and autism-specific features.	Word of mouth and social media	Autistic people (n=11)	Masters dissertation	Semi- structured interviews	Thematic Analysis (Braun and Clarke, 2006).	Person first language Author with Specific Learning Difficulties	Not Specified	Y (9) N (0) CS (1) H

**Table 2:** Themes covered in papers by participant group.

	Autistic People																				Parents and others																			
	Preparation			Education, Skill Development			Personal Awareness			Impact						Management			Support		Preparation			Education, Skill Development			Personal Awareness			Impact						Management			Support	
	Planning	Communication	Ahead of Time	Independence	The Need to Understand	Tailored resources	Not being ready	Puberty as positive	Organising self	Individual Differences	Breaching societal norms	Social experiences	Emotional experiences	Physical experiences	Autism specific	Menstrual products	Support tools	Menstrual Accidents	Wider Support Network	Familial Support	Planning	Communication	Ahead of time	Independence	The Need to understand	Tailored resources	Not being ready	Puberty as positive	Organising self	Individual Differences	Breaching societal norms	Social experiences	Emotional experiences	Physical experiences	Autism specific	Menstrual products	Support tools	Menstrual Accidents	Wider Support Network	Familial Support
Cridland (2014)																						✓		✓							✓				✓	✓				✓
Cummins (2018)																						✓		✓		✓		✓		✓	✓	✓	✓	✓	✓	✓	✓		✓	✓
Eriksen (2016)	✓	✓	✓		✓	✓	✓	✓	✓	✓		✓	✓	✓	✓	✓	✓	✓	✓	✓	✓	✓	✓	✓	✓	✓	✓		✓	✓	✓	✓	✓	✓	✓	✓	✓	✓	✓	✓
Gray & Durand (2023)	✓				✓		✓		✓	✓	✓	✓	✓	✓	✓		✓		✓	✓																				
Grove et al. (2023)								✓		✓			✓	✓	✓	✓																								
Jones (2022)^2^	✓		✓		✓	✓	✓		✓	✓			✓	✓	✓	✓	✓	✓	✓	✓																				
Kendall (2023)^2^										✓		✓	✓	✓	✓																									
Mademtzi (2018)																													✓		✓		✓		✓					
Navot (2017)																						✓					✓				✓		✓		✓		✓			✓
Ross (2017)^2^			✓		✓				✓	✓						✓		✓	✓																					
Steward^1^ (2018)			✓		✓	✓				✓	✓		✓	✓	✓	✓	✓	✓	✓	✓																				
Whiting (2020)	✓	✓	✓		✓	✓	✓	✓	✓	✓	✓	✓	✓	✓	✓	✓	✓	✓	✓	✓																				

1 Non-Autistic participants in this study included parents, professionals and siblings, however, it wasn’t clear which of these groups quotes were attributed to and so weren’t included in this table.

2 Online source. Note: Cells shaded grey are where these participants were not involved in the source.

## Data Availability

This study was a systematic review that did not produce any new data. Accordingly, there is no data to be made available.

## References

[1] Critchley HOD, Babayev E, Bulun SE (2020). Menstruation: science and society. American Journal of Obstetrics and Gynecology.

[2] Barrington DJ, Robinson HJ, Wilson E, Hennegan J (2021). Experiences of menstruation in high income countries: A systematic review, qualitative evidence synthesis and comparison to low- and middle-income countries. PLOS ONE.

[3] Grandi G, Ferrari Xholli (2012). Prevalence of menstrual pain in young women: what is dysmenorrhea?. Journal of Pain Research.

[4] Harder C, Velho RV, Brandes I, Sehouli J, Mechsner S (2024). Assessing the true prevalence of endometriosis: A narrative review of literature data. International Journal of Gynecology &amp; Obstetrics.

[5] Kolovos G, Dedes I, Imboden S, Mueller M (2024). Adenomyosis—A Call for Awareness, Early Detection, and Effective Treatment Strategies: A Narrative Review. Healthcare.

[6] Attia GM, Alharbi OA, Aljohani RM (2023). The Impact of Irregular Menstruation on Health: A Review of the Literature. Cureus.

[7] Modzelewski S, Oracz A, Żukow X, Iłendo K, Śledzikowka Z, Waszkiewicz N (2024). Premenstrual syndrome: new insights into etiology and review of treatment methods. Frontiers in Psychiatry.

[8] Nillni YI, Rasmusson AM, Paul EL, Pineles SL (2021). The Impact of the Menstrual Cycle and Underlying Hormones in Anxiety and PTSD: What Do We Know and Where Do We Go From Here?. Current Psychiatry Reports.

[9] Parker M, Hannah M, Zia A (2022). “If I wasn’t a girl”: Experiences of adolescent girls with heavy menstrual bleeding and inherited bleeding disorders. Research and Practice in Thrombosis and Haemostasis.

[10] McHugh MC (2020). The Palgrave handbook of critical menstruation studies.

[11] Anne Burrows SJ (2006). Girls’ experiences of menarche and menstruation. Journal of Reproductive and Infant Psychology.

[12] Prince HE, Annison E (2023). The impact of menstruation on participation in adventurous activities. Sport, Education and Society.

[13] Michel J, Mettler A, Schönenberger S, Gunz D (2022). Period poverty: why it should be everybody’s business. Journal of Global Health Reports.

[14] Cleghorn E (2021). Unwell Women: Misdiagnosis and Myth in a Man-Made World.

[15] Sebring JCH (2021). Towards a sociological understanding of medical gaslighting in western health care. Sociol Health Illn.

[16] Celik H, Lagro-Janssen TA, Widdershoven GG, Abma TA (2011). Bringing gender sensitivity into healthcare practice: a systematic review. Patient Educ Couns.

[17] Homan P (2019). Structural Sexism and Health in the United States: A New Perspective on Health Inequality and the Gender System. American Sociological Review.

[18] Hudson N (2021). The missed disease? Endometriosis as an example of ‘undone science’. Reproductive Biomedicine & Society Online.

[19] Agarwal P (2022). Hysterical: Exploding the myth of gendered emotions.

[20] Walsh C, O’Connor P, Walsh E, Lydon S (2023). A Systematic Review of Interventions to Improve Healthcare Experiences and Access in Autism. Review Journal of Autism and Developmental Disorders.

[21] Shaw SC, Carravallah L, Johnson M (2024). Barriers to healthcare and a ‘triple empathy problem’ may lead to adverse outcomes for autistic adults: A qualitative study. Autism.

[22] Malik-Soni N, Shaker A, Luck H (2022). Tackling healthcare access barriers for individuals with autism from diagnosis to adulthood. Pediatric Research.

[23] Morris R, Greenblatt A, Saini M (2019). Healthcare Providers’ Experiences with Autism: A Scoping Review. Journal of Autism and Developmental Disorders.

[24] Haney JL (2016). Autism, females, and the DSM-5: Gender bias in autism diagnosis. Social Work in Mental Health.

[25] Moseley RL, Druce T, Turner-Cobb JM (2021). Autism research is ‘all about the blokes and the kids’: Autistic women breaking the silence on menopause. British Journal of Health Psychology.

[26] Green RM, Travers AM, Howe Y, McDougle CJ (2019). Women and Autism Spectrum Disorder: Diagnosis and Implications for Treatment of Adolescents and Adults. Current Psychiatry Reports.

[27] Anderson-Chavarria M (2021). The autism predicament: models of autism and their impact on autistic identity. Disability & Society.

[28] Lai M-C, Szatmari Petera (2020). Sex and gender impacts on the behavioural presentation and recognition of autism. Current Opinion in Psychiatry.

[29] Lockwood Estrin G, Milner V, Spain D, Happé F, Colvert E (2021). Barriers to Autism Spectrum Disorder Diagnosis for Young Women and Girls: a Systematic Review. Review Journal of Autism and Developmental Disorders.

[30] Groenman AP, Torenvliet C, Radhoe TA, Agelink van Rentergem JA, Geurts HM (2022). Menstruation and menopause in autistic adults: Periods of importance?. Autism.

[31] Grant A, Jones S, Williams K, Leigh J, Brown A (2022). Autistic women’s views and experiences of infant feeding: A systematic review of qualitative evidence. Autism.

[32] Steward R, Crane L, Mairi Roy E, Remington A, Pellicano E (2018). “Life is Much More Difficult to Manage During Periods”: Autistic Experiences of Menstruation. J Autism Dev Disord.

[33] Babbar K, Martin J, Ruiz J, Parray AA, Sommer M (2022). Menstrual health is a public health and human rights issue. The Lancet Public Health.

[34] Hamilton A, Marshal MP, Murray PJ (2011). Autism spectrum disorders and menstruation. J Adolesc Health.

[35] Perlow EJI (2019). Adult Women with and Without Autism Spectrum Disorder: Perspectives on the Menarche/menstruation Experience.

[36] Ibrahim I (2021). Autism Spectrum Disorder, Premenstrual Symptoms, and Premenstrual Dysphoric Disorder.

[37] Page MJ, McKenzie JE, Bossuyt PM (2021). The PRISMA 2020 statement: an updated guideline for reporting systematic reviews. BMJ.

[38] Campisi SC, Marchand JD, Siddiqui FJ, Islam M, Bhutta ZA, Palmert MR (2020). Can we rely on adolescents to self-assess puberty stage? A systematic review and meta-analysis. The Journal of Clinical Endocrinology &amp; Metabolism.

[39] Li W, Liu Q, Deng X, Chen Y, Liu S, Story M (2017). Association between Obesity and Puberty Timing: A Systematic Review and Meta-Analysis. International Journal of Environmental Research and Public Health.

[40] Armour M, Parry K, Al-Dabbas MA (2019). Self-care strategies and sources of knowledge on menstruation in 12,526 young women with dysmenorrhea: A systematic review and meta-analysis. PLOS ONE.

[41] Ellis R, Williams K, Brown A, Healer E, Grant A (2023). A realist review of health passports for Autistic adults. PLOS ONE.

[42] Evans RL, Harris B, Onuegbu C, Griffiths F (2022). Systematic review of educational interventions to improve the menstrual health of young adolescent girls. BMJ Open.

[43] Programme CAS CASP Checklists.

[44] Woodman C, Baillie J, Sivell S (2016). The preferences and perspectives of family caregivers towards place of care for their relatives at the end-of-life. A systematic review and thematic synthesis of the qualitative evidence. BMJ Supportive &amp; Palliative Care.

[45] Thomas J, Harden A (2008). Methods for the thematic synthesis of qualitative research in systematic reviews. BMC Medical Research Methodology.

[46] Fryer T (2022). A critical realist approach to thematic analysis: producing causal explanations. Journal of Critical Realism.

[47] Zhang T (2023). Critical Realism: A Critical Evaluation. Social Epistemology.

[48] Williams GL, Ellis R, Holloway W (2024). ‘Building our own house’ as an insider-only Community-Partnered Participatory Research Council: Co-creating a safe space for Autistic knowledge production. Autism.

[49] Lumivero (2020). NVivo.

[50] Gray LJ, Durand H (2023). Experiences of dysmenorrhea and its treatment among allistic and autistic menstruators: a thematic analysis. BMC Womens Health.

[51] Grove R, Clapham H, Moodie T, Gurrin S, Hall G (2023). ‘Living in a world that’s not about us’: The impact of everyday life on the health and wellbeing of autistic women and gender diverse people. Womens Health (Lond).

[52] Eriksen WT (2016). Facing Puberty: Exploring The Onset, Symptoms And Experience Of Menses In Females With Autism Spectrum Disorder.

[53] Ross K (2017). How I Learned To Love Having My Period as an Autistic Woman − Kate Ross. https://www.spectrumwomen.com/wellbeing/how-i-learned-to-love-having-my-period-as-an-autistic-woman-kate-ross/.

[54] Kendall K (2023). This Is Why PMS Sucks for Autistic Women. https://kirstykendall.com/pms-autistic-women/.

[55] Jones SaD S. How people on the autism spectrum experience and manage their periods A report by INTIMINA. https://www.intimina.com/blog/wp-content/uploads/2022/03/Intimina-Autism-Report-V1f.pdf.

[56] Cridland EKJS, Caputi P, Magee CA (2014). Being a girl in a boys’ world: investigating the experiences of girls with autism spectrum disorders during adolescence. J Autism Dev Disord.

[57] Cummins C, Pellicano E, Crane L (2020). Supporting Minimally Verbal Autistic Girls with Intellectual Disabilities Through Puberty: Perspectives of Parents and Educators. J Autism Dev Disord.

[58] Mademtzi M, Singh P, Shic F, Koenig K (2018). Challenges of Females with Autism: A Parental Perspective. J Autism Dev Disord.

[59] Navot N, Jorgenson AG, Webb SJ (2017). Maternal experience raising girls with autism spectrum disorder: a qualitative study. Child Care Health Dev.

[60] Whiting V (2020). The Experiences of Puberty in Autism Spectrum Condition: Retrospective reflections from women on the Autism Spectrum.

[61] Botha M, Dibb B, Frost DM (2022). “Autism is me”: an investigation of how autistic individuals make sense of autism and stigma. Disability &amp; Society.

[62] Chapman R, Carel H (2022). Neurodiversity, epistemic injustice, and the good human life. Journal of Social Philosophy.

[63] Sinclair J (1993). Don’t Mourn For Us. Our Voice.

[64] Botha M, Cage E (2022). “Autism research is in crisis”: A mixed method study of researcher’s constructions of autistic people and autism research. Frontiers in Psychology.

[65] Ballan MS (2012). Parental Perspectives of Communication about Sexuality in Families of Children with Autism Spectrum Disorders. Journal of Autism and Developmental Disorders.

[66] Steele L, Goldblatt B (2020). The Human Rights of Women and Girls with Disabilities: Sterilization and Other Coercive Responses to Menstruation.

[67] Michielsen K, Brockschmidt L (2021). Barriers to sexuality education for children and young people with disabilities in the WHO European region: a scoping review. Sex Education.

[68] Bennett C, Harden J (2014). An exploration of mothers’ attitudes towards their daughters’ menarche. Sex Education.

[69] Koegel LK, Detar WJ, Fox A, Koegel RL (2014). Adolescents and Adults with Autism Spectrum Disorders.

[70] Walsh C, Lydon S, O’Dowd E, O’Connor P (2020). Barriers to Healthcare for Persons with Autism: A Systematic Review of the Literature and Development of A Taxonomy. Dev Neurorehabil.

[71] Doherty AJ, Atherton H, Boland P (2020). Barriers and facilitators to primary health care for people with intellectual disabilities and/or autism: an integrative review. BJGP Open.

[72] Memarian A MS (2015). Therapeutic and Ethical Dilemma of Puberty and Menstruation Problems in an Intellectually Disabled (Autistic) Female: a Case Report. Acta Med Iran.

[73] Kinnaird E, Stewart C, Tchanturia K (2019). Investigating alexithymia in autism: A systematic review and meta-analysis. European Psychiatry.

[74] Liu J, Chen LL, Shen S (2020). Challenges in the Diagnosis and Management of Pain in Individuals with Autism Spectrum Disorder. Review Journal of Autism and Developmental Disorders.

[75] Garcia-Villamisar D, Moore D, Garcia-Martínez M (2019). Internalizing Symptoms Mediate the Relation Between Acute Pain and Autism in Adults. Journal of Autism and Developmental Disorders.

[76] Mason D, Ingham B, Urbanowicz A (2019). A Systematic Review of What Barriers and Facilitators Prevent and Enable Physical Healthcare Services Access for Autistic Adults. Journal of Autism and Developmental Disorders.

[77] Lese K (2016). Padded assumptions: A critical discourse analysis of patriarchal menstruation discourse.

[78] Costos D, Ackerman R, Paradis L (2002). Sex Roles.

[79] Morgan H, Durman E, Henry K, Jones C, Byrom S, Lawson WB (2024). Supporting Autistic People Through Pregnancy and Childbirth.

[80] Grant A, Griffiths C, Williams K, Brown A (2024). “It felt like I had an old fashioned telephone ringing in my breasts”: An online survey of UK Autistic birthing parents’ experiences of infant feeding. Maternal &amp; Child Nutrition.

[81] Brown N, Williams R, Bruinvels G, Piasecki J, Forrest LJ (2022). Teachers’ Perceptions and Experiences of Menstrual Cycle Education and Support in UK Schools. Frontiers in Global Women’s Health.

[82] UK PI (2018). Break The Barriers: Girls’ Experiences of Menstruation in the UK.

[83] Mondragon NI, Txertudi MB (2019). Understanding menstruation: Influence of gender and ideological factors. A study of young people’s social representations. Feminism & Psychology.

[84] Moyse R, Porter J (2015). The experience of the hidden curriculum for autistic girls at mainstream primary schools. European Journal of Special Needs Education.

[85] Rubinsky V, Gunning JN, Cooke-Jackson A (2020). “I Thought I Was Dying:” (Un)Supportive Communication Surrounding Early Menstruation Experiences. Health Communication.

[86] Hannah LA, Stagg SD (2016). Experiences of Sex Education and Sexual Awareness in Young Adults with Autism Spectrum Disorder. Journal of Autism and Developmental Disorders.

[87] Björnsdóttir K, Stefánsdóttir GV (2020). Double Sexual Standards: Sexuality and People with Intellectual Disabilities Who Require Intensive Support. Sexuality and Disability.

[88] Hennegan J, Winkler IT, Bobel C (2021). Menstrual health: a definition for policy, practice, and research. Sexual and Reproductive Health Matters.

[89] Kohn BH, Vidal P, Chiao R, Pantalone DW, Faja S (2023). Sexual Knowledge, Experiences, and Pragmatic Language in Adults With and Without Autism: Implications for Sex Education. J Autism Dev Disord.

[90] Nicolaidis C, Kripke CC, Raymaker D (2014). Primary Care for Adults on the Autism Spectrum. Medical Clinics of North America.

[91] Christina Nicolaidis DR, Kapp Steven K, Baggs Amelia, McDonald Katherine, Weiner Michael, Maslak Joelle, Hunter Morrigan, Joyce Andrea (2019). The AASPIRE practice-based guidelines for the inclusion of autistic adults in research as co-researchers and study participants. Autism.

